# Feasibility/acceptability of an acceptance‐based therapy intervention for diverse adolescent girls with overweight/obesity

**DOI:** 10.1002/osp4.483

**Published:** 2021-03-01

**Authors:** Michelle I. Cardel, Alexandra M. Lee, Xiaofei Chi, Faith Newsome, Darci R. Miller, Angelina Bernier, Lindsay Thompson, Matthew J. Gurka, David M. Janicke, Meghan L. Butryn

**Affiliations:** ^1^ Department of Health Outcomes and Biomedical Informatics University of Florida College of Medicine Gainesville Florida USA; ^2^ Center for Integrative Cardiovascular and Metabolic Diseases University of Florida Gainesville Florida USA; ^3^ Department of Pediatrics University of Florida College of Medicine Gainesville Florida USA; ^4^ Department of Clinical and Health Psychology University of Florida College of Public Health and Health Professions Gainesville Florida USA; ^5^ Department of Psychology and Center for Weight Eating and Lifestyle Science Drexel University College of Arts and Sciences Philadelphia Pennsylvania USA

**Keywords:** adolescents, feasibility, intervention, obesity

## Abstract

**Background:**

Behavioral obesity interventions using an acceptance‐based therapy (ABT) approach have demonstrated efficacy for adults, yet feasibility and acceptability of tailoring an ABT intervention for adolescents remains unknown.

**Objective:**

This study assessed the feasibility and acceptability of an ABT healthy lifestyle intervention among diverse adolescent cisgender girls with overweight/obesity (OW/OB).

**Methods:**

Adolescent cisgender girls aged 14–19 with a BMI of ≥85th percentile‐for‐sex‐and‐age were recruited for participation in a single‐arm feasibility study. The primary outcomes were recruitment and retention while the secondary outcome was change in BMI Z‐score over the 6‐month intervention. Exploratory outcomes included obesity‐related factors, health‐related behaviors, and psychological factors.

**Results:**

Recruitment goals were achieved; 13 adolescents (>60% racial/ethnic minorities) participated in the intervention, and 11 completed the intervention (85% retention). In completers (*n* = 11), a mean decrease in BMI Z‐score of −0.15 (*SD* = 0.34, Cohen's *d* = −0.44) was observed. Improvements were also noted for change in percentage of 95th percentile (*d* = −0.35), percent body fat (*d* = −0.35), quality of life (*d* = 0.71), psychological flexibility (*d* = −0.86), and depression (*d* = −0.86).

**Conclusions:**

These preliminary findings suggest an ABT healthy lifestyle intervention tailored for adolescent cisgender girls with OW/OB may be an acceptable treatment that could lead to improvements in BMI Z‐score, obesity‐related measures, and psychological outcomes.

## 1 INTRODUCTION

Prevalence of overweight/obesity (OW/OB) among US adolescents has reached 34.5%, and obesity prevalence is highest among Hispanic and non‐Hispanic Black adolescent females.^1^ From 1999 to 2016, adolescent females experienced significant increases in prevalence of overweight and all three classes of obesity,^1^ making adolescent females, particularly those from racial/ethnic minority groups where health disparities often stem from social determinants of health and structural racism, vulnerable to the adverse physical, social, and mental health complications resulting from obesity.^2^, ^3^ Efforts to improve treatment efficacy for adolescent weight loss have generally been disappointing,^4^, ^5^, ^6^, ^7^ as the amount of weight lost is often limited, weight regain is common, and compliance to treatment has been difficult to quantify.^2^, ^3^, ^4^, ^5^, ^6^, ^7^, ^8^

Comprehensive lifestyle interventions are the first line of treatment for adolescents with OW/OB.^2^, ^3^ Standard behavioral therapy (SBT) interventions typically include (1) use of structured goals for reducing calorie intake and increasing physical activity; (2) regular self‐monitoring of weight, eating, and physical activity; (3) monitoring of weight and progress by an instructor to provide updated feedback, support, and enhanced accountability; and (4) additional behavioral skills training such as goal‐setting, problem solving, and stimulus control.^9^

Adolescent SBT interventions have demonstrated modest results,^2^, ^3^ potentially due to little focus on self‐regulation skills and psychological tools necessary to initiate and maintain health‐related behaviors for weight management. Acceptance‐based therapy (ABT) integrates evidence‐based components of SBT with acceptance and commitment therapy (ACT).^9^, ^10^, ^11^ The goal of ABT is to promote willingness to engage in behaviors that are consistent with long‐term goals and values, even when doing so is challenging. ABT teaches the ability to tolerate (accept) uncomfortable experiences that are a natural part of weight loss (e.g., cravings, desire to be sedentary), and allows for identification and internalization of values, and ongoing commitment to behavior that is consistent with those values.^9^, ^10^, ^11^ Such strategies can attenuate the waning of commitment and return to previous (automatic) decision‐making processes often observed among adolescents in SBT interventions. Adolescence is characterized by changes in emotional and mental development that increase impulsivity and inhibit self‐regulation and executive function.^12^ It is also a life stage that includes notable improvement of cognitive learning abilities and flexibility for adjustment of goals and priorities,^13^ thus skills learned with ABT may be particularly relevant for adolescents.

Among adults, a network meta‐analysis found that ABT interventions had the most consistent evidence of effectiveness for weight loss compared to SBT interventions at postintervention and follow‐up.^14^ Unlike other behavioral interventions, where non‐Hispanic Black participants lose approximately 50% less weight than non‐Hispanic whites,^15^, ^16^ adult weight loss with ABT did not differ by race, sex, or education.^15^ Furthermore, variations of acceptance‐based interventions have proven successful for treating other medical/behavioral issues in adolescent cisgender girls including high‐risk sexual behavior, anorexia, and depression.^17^, ^18^, ^19^ Moreover, ACT, when used in conjunction with lifestyle modifications, has demonstrated to be an acceptable family‐based intervention that improves weight, cognitive restraint, hunger, physical activity, and psychosocial outcomes in adolescents with obesity.^20^

Feasibility and acceptability of an ABT lifestyle intervention that focuses primarily on the adolescent (rather than heavily focusing on parents) and is tailored to the needs of adolescent cisgender girls with OW/OB remains unknown. Given the success of ABT observed for weight loss in adults across diverse populations and in adolescents with other complex behaviors and disease states, utilization of ABT in adolescents with OW/OB could have significant effects on weight and cardiometabolic risk.

This feasibility study investigated the acceptability of a 6‐month ABT healthy lifestyle intervention in adolescent cisgender girls aged 14–19 with OW/OB. Primary outcomes included recruitment and retention; a secondary outcome was change in BMI Z‐score at postintervention. Exploratory outcomes including health‐related behaviors and psychological outcomes were also assessed.

## 2 METHODS

### 2.1 Participants

Adolescent cisgender girls aged 14 –19 were recruited via flyers, social media, and local pediatric clinics. An integrated data repository, a large‐scale database that organizes information from clinical and research enterprises, was also used to identify potential participants. Once interest was indicated, potential participants were screened for eligibility. For study inclusion, participants had a BMI ≥85th percentile‐for‐sex‐and‐age based on Centers for Disease Control and Prevention (CDC) growth charts.^21^ Exclusion criteria included diagnosed diabetes (Type I or II), any major health condition (i.e., active or chronic infections, cancer, cardiovascular disease, kidney disease, or lung disease), any condition prohibiting physical activity, diagnosed eating disorders, recent or ongoing substance abuse disorders, weight loss ≥5% in the previous 6 months, or those who had begun or changed the dosage of any medication known to affect appetite or body composition within the previous 3 months.

### 2.2 Protocol

After initial screening, participants were scheduled for a baseline clinic visit. Anthropometrics, percent body fat, and blood pressure measurements were assessed, and participants were asked to complete a series of questionnaires. Participants were given an accelerometer to objectively measure physical activity for a 7‐day period, then scheduled for a second clinic visit, which included a nutrition consultation with a registered dietitian and completion of additional surveys. Between the first and second clinic visits, participants completed three dietary recalls at random including one weekend day and two weekdays.

Once both clinic visits were complete, participants were invited to join a single‐arm, 6‐month ABT healthy lifestyle intervention that included 15 group‐based sessions, each approximately 90 min in length: weekly for 2 months, biweekly for 2 months, and monthly for 2 months, for a total of 22.5 contact hours in sessions. Between Sessions 13 and 14, and Sessions 14 and 15, participants completed a phone check‐in, given the length of time between sessions. Throughout the intervention, participants were invited to partake in an optional GroupMe® (GroupMe, Inc.) chat monitored regularly by the research team to facilitate continued engagement, support, and comradery outside of intervention sessions. Session absences were permitted to be made up and counted toward session attendance by meeting one‐on‐one, in‐person with the instructor, as adolescents in formative work cited flexibility as necessary to remain engaged.^22^

Upon completing the 6‐month intervention, a postintervention clinic visit was conducted to obtain anthropometrics, percent body fat, blood pressure, and responses to questionnaires for a final time. Final dietary recalls were collected at random (one weekend day and two weekdays) and accelerometers were given to participants to collect physical activity measurements for 7 days.

During a portion of this protocol, the 2020 COVID‐19 pandemic factored into implementation.^22^ Four participants transitioned from in‐person to remote intervention delivery for sessions nine through 15 of the WATCH© intervention. The ability to conduct postintervention clinic visits was limited, thus two participants did not attend a postintervention clinic visit, but completed all surveys and dietary recalls, wore accelerometers, and utilized WiFi scales to objectively measure weight. Institutional Review Board approval (IRB201701609; NCT03284788) was obtained.

### 2.3 ABT healthy lifestyle intervention

**FIGURE 1:**
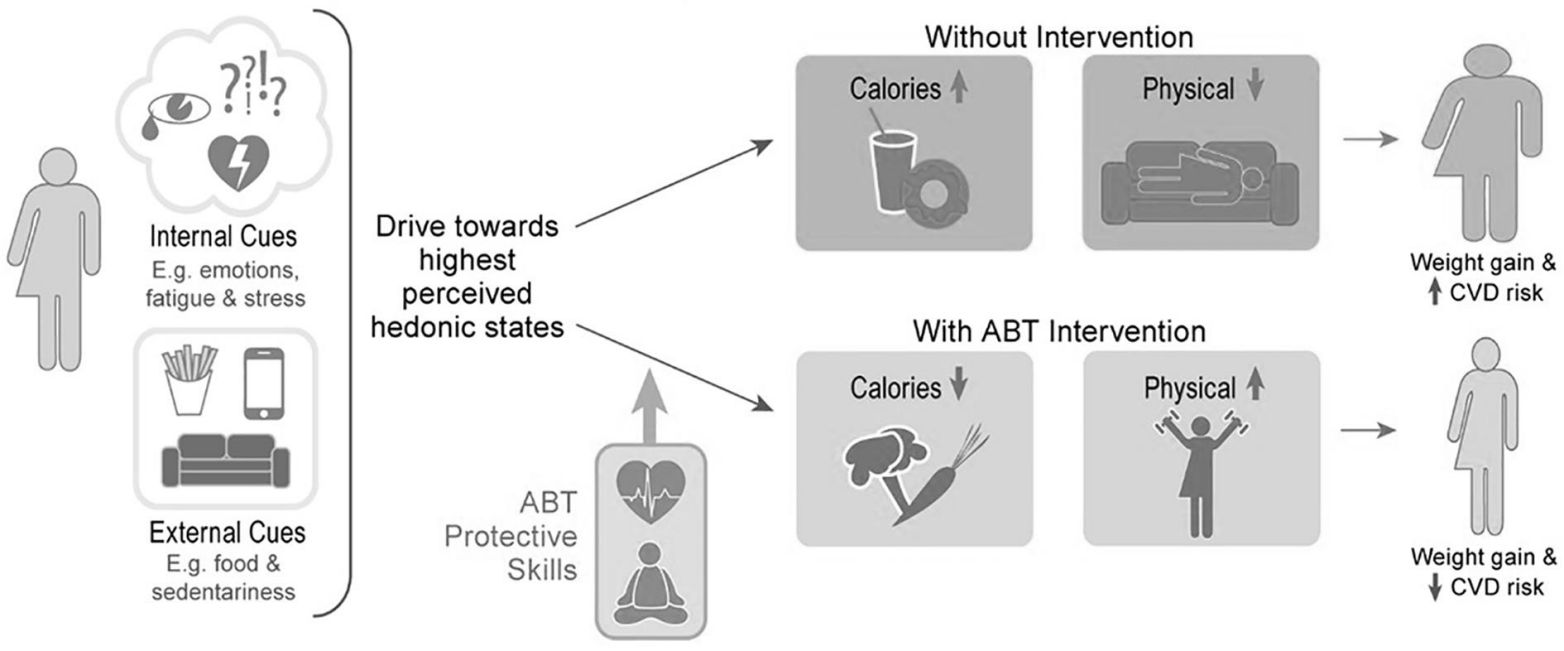
Conceptual model of proposed protective effects of ABT on adolescent body weight and CVD risk. In the conceptual model, physiological drivers generate a “hedonic treadmill,” which is characterized by a chronic desire for highly palatable food and conservation of energy. These drivers are activated by our current obesogenic environment, with an omnipresence of food stimuli and availability, and encouragement of physical inactivity (e.g., labor‐saving devices). Coupled with internal cues (e.g., stress), the physiological response is a desire to reach a higher perceived hedonic state via consumption of highly palatable, energy dense food, and sedentary behavior. These drives often result in choices at odds with an adolescent's goals, including dietary and physical activity lapses. Thus, unless self‐regulation skills are learned and utilized, and ability to engage in behaviors consistent with their values and goals despite uncomfortable thoughts, feelings, or cravings, excess energy consumption, and sedentary activity can become default behaviors, increasing an adolescent's risk for CVD over a lifespan. Others have demonstrated ABT effectiveness for adult weight loss, and we posit and demonstrate preliminary evidence that ABT is a feasible and acceptable intervention for adolescent girls with OW/OB. An ABT intervention has the potential to be effective in reducing excess adiposity among adolescent girls. ABT includes a focus on self‐regulation skills to improve: (1) tolerance of uncomfortable internal states (e.g., anxiety, stress) and perceived reduction of pleasure (e.g., choosing physical activity instead of watching TV); (2) behavioral commitment to clearly defined values, which will increase motivation to maintain difficult weight‐control behaviors; and (3) metacognitive awareness of decision‐making. Developing these self‐regulation skills is posited to be protective against dietary and physical activity lapses, leading to negative energy balance, decreased weight, and improved CVD risk in adolescents over the long‐term

The intervention curriculum, or Wellness Achieved Through Changing Habits (WATCH©), was developed using an adolescent‐engaged, iterative mixed methods approach in which materials from adult ABT interventions were used as a conceptual guide.^23^ Adult ABT weight management interventions incorporate key elements of ACT and integrate these into evidence‐based SBT weight management treatments (SBT + ACT). This approach retains core behavioral components of SBT including (1) structured goals for reducing calorie intake and increasing physical activity; (2) regular self‐monitoring; (3) instructor feedback/support to enhance accountability; and (4) behavioral skills training such as stimulus control.^9^ ACT strategies integrated into our ABT curriculum include (1) acceptance‐based strategies and willingness to face uncomfortable internal experiences (e.g., cravings, emotions); (2) values clarification and ongoing commitment to values‐based actions; (3) cognitive defusion, or the ability to “step back and detach”; and (4) mindful decision making. The conceptual model for ABT's potential effects on weight and cardiovascular disease (CVD) risk among adolescents is included in Figure 1 (modified from Forman and Butryn).^23^

Once the WATCH© intervention was developed, an additional iterative process involved focus group discussions^25^, ^26^ to further tailor the curriculum to be developmentally appropriate and relevant for adolescents. These discussions captured preferences of adolescents with OW/OB for a behavioral weight loss intervention including messaging, content, and ideal methods for engagement. Through this process, important developments regarding delivery and messaging were implemented. The expressed need for cisgender‐specific interventions among focus group participants was considered, resulting in the delivery of the intervention herein to adolescent cisgender girls. Further, the messaging was shifted from weight loss to healthy lifestyles when possible. The expertise of adolescent citizen scientists, adolescents who provided their feedback and expertise based on the lived experience of being an adolescent with OW/OB, was also utilized collaboratively to revise the intervention to be acceptable and relevant to adolescent cisgender girls with OW/OB.

All 15 group sessions followed a similarly organized format over the 90‐min session: individual check‐ins (including optional weigh‐ins; 10 min); group physical activity (15 min); review of key points, goals, and accomplishments from the previous session (10 min); new content (45 min); healthy snack (5 min); and goal‐setting for the next session (5 min). Parents/guardians were welcomed to optionally participate in three sessions: Session 3 (grocery store tour), Session 6 (handling difficult environmental/social situations; how to discuss weight and provide support), and Session 13 (supporting over the long term). As this intervention includes adolescents in both the adult (18+) and child (17 and under) age ranges, parent/guardian participation was not always applicable and thus, not all parents/guardians participated in the optional sessions. The lead instructor (Michelle I. Cardel) was the Principal Investigator (PI) of the study, who holds a doctorate degree in nutrition sciences and is a registered dietitian trained in lifestyle counseling, implementation science, and ABT.

### 2.4 Primary outcomes

The primary outcomes were measures of recruitment and retention. A priori benchmarks of acceptable measures of recruitment and retention included: (1) recruiting fifteen 15 cisgender girls aged 14–19 to enroll in the study within an 8‐week period (over two study cohorts) by providing consent/assent and completing both initial baseline clinic visits; (2) at least 80% of enrolled participants deciding to participate in the intervention; (3) ≥70% of intervention completers attending all 15 study sessions, and retention of more than 50% of intervention participants completing the 6‐month intervention and postintervention clinical assessment. These benchmarks were selected based on previous research,^3^, ^4^, ^6^, ^20^, ^26^ including studies demonstrating attrition rates in youth obesity interventions range between 20% and 40%.^27^

### 2.5 Physiological measures

Height was measured to the nearest 0.1 cm using a wall‐mounted stadiometer (Holtain Limited Harpende; Veeder‐Root,) with shoes removed. Participants were weighed to the nearest 0.1 kg on a digital scale (Health O Meter 2600KL Wheelchair Scale). The CDC SAS program was used to calculate BMI Z‐score, based upon the 2000 CDC growth charts.^28^ The secondary outcome was a clinically significant change in BMI Z‐score ≥−0.15, as this decrease has been shown to lead to improvements in ≥1 CVD risk factor.^29^

Additional physiological markers were exploratory outcomes. Percentage of the 95th percentile was also calculated based upon the 2000 CDC growth charts, an analytical approach recommended for use when characterizing particularly high BMI values in adolescents.^30^ Percent body fat was assessed using BodPod (Life Measurement, Inc.), a reliable and valid system for measuring adolescent body composition using air displacement plethysmography.^31^ Systolic and diastolic blood pressure measurements were obtained with an automated monitor (Omron BP710N; Omron Europe) in duplicate with 2 min between each measure, then averaged.

### 2.6 Health‐related behavioral measures

Health‐related behavioral changes were included as exploratory outcome variables. Dietary data were collected via phone by a trained research assistant using the National Cancer Institute (NCI) automated self‐administered 24‐h dietary recall system (ASA‐24). The ASA‐24 is based on the Automated Multiple Pass Method, demonstrating high validity and reliability as well as decreased reports of underreporting food intake when compared to other methods of dietary data collection.^32^, ^33^ Diet quality was calculated using the Healthy Eating Index (HEI) for participants' general intake. SAS code for the HEI calculations (2015 version) was developed using sample code from the NCI and the US Department of Agriculture.^34^

Activity data were collected with an Actigraph wGT3X‐BT (Actigraph LLC) accelerometer worn on participants' nondominant wrist for 7 days. Participants were instructed to wear the monitor at all times, except during activities involving direct contact with water that could result in monitor submersion. Monitors were initialized with a sampling rate of 100 Hz and raw .gt3x data were downloaded and converted to a. csv using the ActiLife software (ActiLife 6.13.4; Actigraph LLC). Raw accelerometer data were processed with GGIR (https://CRAN.R‐project.org/package=GGIR) to identify average minutes per day spent in moderate‐to‐vigorous physical activity (MVPA) and minutes inactive using thresholds adapted for an adolescent population.^35^ Sleep was differentiated from, and not counted toward, total average inactive minutes per day.^36^ To be included in analysis, participants needed three days of activity data with at least 10 daytime hours recorded daily.

Sleep duration was identified in part four of the GGIR output using the HCDZA guider, validated by van Hees et al.^36^ To be included in analysis, participants needed ≥3 valid nights of data. Average sleep duration was calculated as the difference between number of hours at sleep onset and sleep wake across all valid nights.

### 2.7 Psychological factors

Psychological factors were included as exploratory outcomes. Perceived quality of life (QOL) was measured using the 23‐item Pediatric Quality of Life Inventory (PedsQL 4.0), which captures health‐related QOL using a five‐point Likert scale. Questions included “In the past ONE month, how much of a problem has this been for you…” (0–4 with 0 = never and 4 = almost always) in four areas (physical, emotional, social, school). Higher scores indicate better health‐related QOL, and acceptable internal consistency and validity among adolescents using PedsQL 4.0 is reported.^37^

Psychological flexibility, rooted in constructs of ABT such that one's behavior aligns with values‐based choice, and experiential avoidance, defined as making choices that avoid difficult thoughts or feelings associated with a particular behavior, were assessed as a process measure of ABT using the Acceptance and Action Questionnaire for Weight‐Related Difficulties (AAQW).^38^ This measure contains 22 questions using a seven‐point Likert scale to assess experiential avoidance or inflexibility in terms of weight‐related difficulties. Lower scores indicate lower experiential avoidance and higher psychological flexibility with total possible scores ranging from 22 to 154. The AAQW has good internal consistency and convergent validity,^38^ and has been validated in older adolescents and racial/ethnic minorities.^39^

Depression levels were measured with the 21‐item Beck Depression Inventory‐II (BDI‐II), using a four‐point ordinal, categorical scale for each item.^40^ Measure scores are summed to create a total that is classified into low, moderate, and significant depression and scores of ≥30 indicate severe clinical depression. BDI‐II is reliable and valid for use in adolescents.^40^

Subjective stress was assessed using the 10‐item Cohen Perceived Stress Scale (PSS‐10). This measure was designed for use in older adolescents and adults to assess the extent to which one's life is unpredictable, uncontrollable, and overwhelming using a five‐point Likert scale to capture the frequency of a participant's feelings and thoughts during the past month (0–4 with 0 = never and 4 = very often). Higher scores indicate greater perceived stress and PSS‐10 demonstrates adequate internal reliability and construct validity.^41^

### 2.8 Statistical analyses

Feasibility and acceptability outcomes were descriptive in nature. Given this was a feasibility study, the study was not powered to detect statistical significance, and analyses reported are based on effect sizes. Cohen's *d* (and 95% confidence intervals) statistics were calculated to provide standardized effect sizes for changes over time for the secondary outcome (BMI Z‐score) and exploratory outcomes. Obesity‐related measures, health‐related behaviors, and psychological factors were assessed among completers using descriptive statistics at baseline, postintervention, and the change from baseline to postintervention. All statistical analyses were conducted using SAS 9.4 (SAS Institute).

## 3 RESULTS

### 3.1 Demographics

**FIGURE 2:**
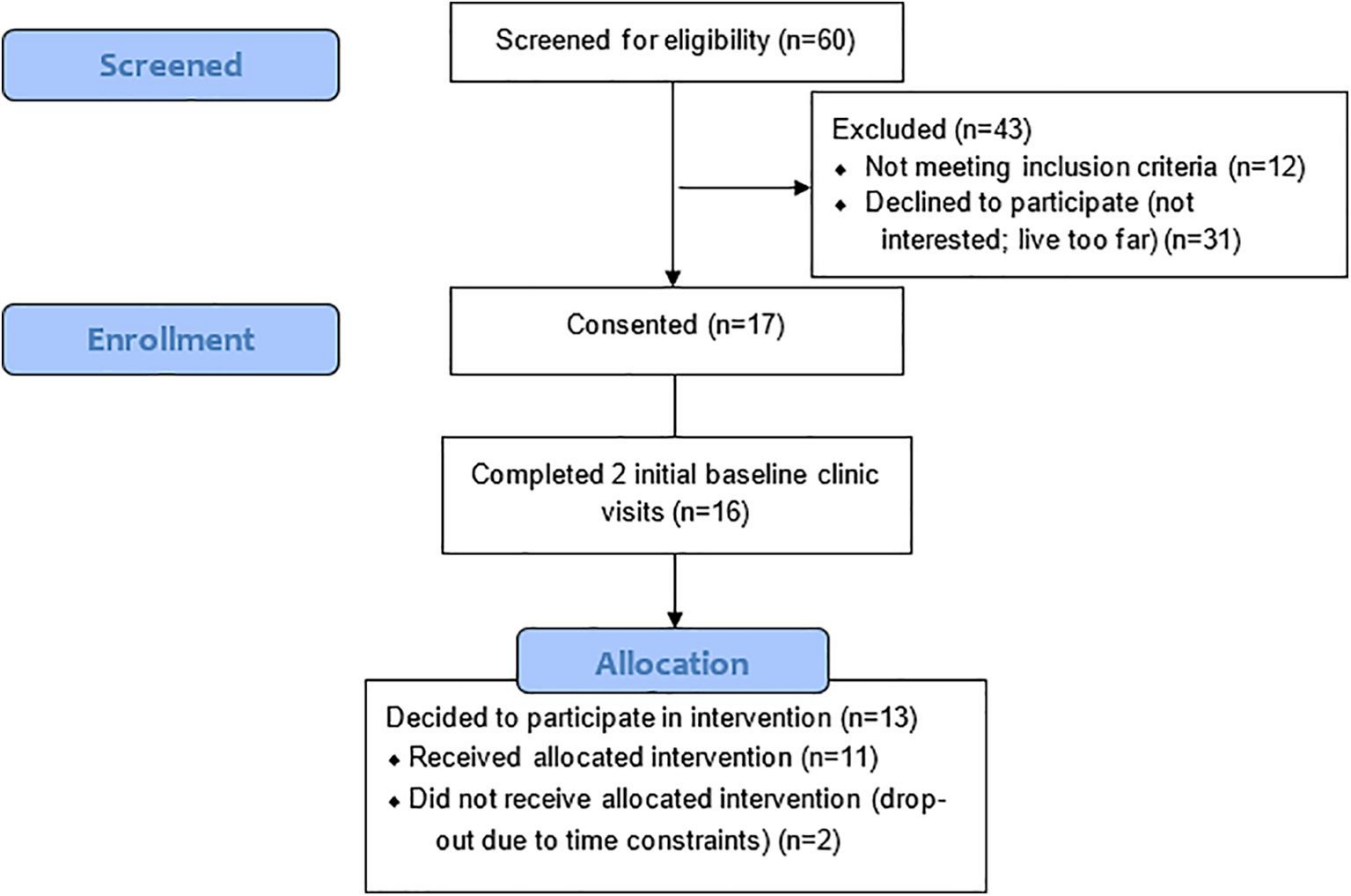
Participant flow

Figure 2 demonstrates participant flow and Table 1 includes demographics and baseline characteristics for intervention participants (*n* = 13) and completers (*n* = 11) in 2019–2020. Mean age was 16.69 ± 1.65 for intervention participants and 16.73 ± 1.68 for completers. Baseline BMI for intervention participants was 35.28 ± 5.5 kg/m^2^ and 35.04 ± 5.58 kg/m^2^ for completers, respectively. Six of thirteen intervention participants (46.2%) and five of eleven completers (45.4%) self‐identified as racial minorities, and two intervention participants and completers self‐identified as Hispanic/Latina. Approximately one‐third of intervention participants and completers reported their highest level of parent education as a graduate or professional degree.

**TABLE 1:** Demographics

Variable	All intervention participants	Completers
	(*n* = 13)	(*n* = 11)
	*N*; % (mean, *SD*)	*N*; % (mean, *SD*)
Age (in years)	16.69 (1.65)	16.73 (1.68)
Race	7 (53.85%)5 (38.46%)1 (7.69%)	6 (54.55%)4 (36.36%)1 (9.09%)
White		
Black		
Multiple races		
Ethnicity	2 (15.38%)11 (84.62%)	2 (18.18%)9 (81.82%)
Hispanic/Latina		
Not Hispanic/Latina		
Highest parental education		1 (9.09%)3 (27.27%)3 (27.27%)4 (36.36%)0 (0%)
High school diploma/GED	2 (15.38%)	
Some college/associate degree	3 (23.08%)	
College graduate	3 (23.08%)	
Graduate or professional degree	4 (30.77%)	
Unknown	1 (7.69%)	
BMI	35.28 (5.50)	35.04 (5.58)

### 3.2 Primary outcomes

A priori benchmarks for determining intervention acceptability were identified and are presented in comparison to study results in Table 2. Sixteen adolescent females (aged 14–19) completed study enrollment and both initial baseline clinic visits. Thirteen (81.3%) eligible participants then decided to participate in the healthy lifestyle intervention. Of these 13 participants, two dropped out due to time constraints (84.6% retention; 15.4% attrition). Of the completers (*n* = 11), 90.9% attended all 15 intervention sessions and the postintervention clinic visit.

**TABLE 2:** Primary and Secondary outcomes: a priori benchmarks versus feasibility study outcomes regarding recruitment and retention

Variable	A priori benchmark	Study results
Enrollment w/in 8‐week period (two cohorts)	*N* = 15	*N* = 16
Percent of enrollees who decide to participate in the intervention	80%	81.3%
Retention of intervention participants at postintervention	>50%	84.6%
Intervention completers session attendance at all 15 sessions	≥70%	90.9%
BMI Z‐score	Clinically significant reduction by ≥−0.15^a^	−0.15 ± 0.34

^a^ Has been shown to lead to improvements in ≥1 CVD risk factor.^29^

### 3.3 Secondary outcome

Results in Table 3 represent measures at baseline, postintervention, changes from baseline to postintervention, and Cohen's *d* for the 11 intervention completers. At baseline, the range of BMI Z‐score (*n* = 13) was 1.33–2.57, and at posttreatment the BMI Z‐score range (*n* = 11) was 0.23–2.59. Intervention completers demonstrated a mean BMI Z‐score loss (secondary outcome) of −0.15 ± 0.34 (95% CI (−0.37, 0.08), *d* = −0.44 (−0.73, −0.14)), though only three out of the 11 completers met the BMI Z‐score reduction goal of ≥−0.15.

**TABLE 3:** Changes in obesity‐related factors, health‐related behaviors, and psychological factors among intervention completers (*n* = 11)

Variable	Baseline (mean, *SD*)	Post‐intervention (mean, *SD*)	Change from baseline to vpostintervention (mean, SD, 95% CI)	Cohen's *d* (95% CI)
Obesity‐related factors				
Weight (kg)	94.07 (15.42)	93.59 (19.02)	−0.48 (5.63), (−4.26, 3.30)	−0.09 (−0.24, 0.07)
Height (cm)	163.78 (4.15)	164.18 (4.45)	0.40 (0.79), (−0.13, 0.93)	0.50 (0.26, 0.74)
BMI Z‐score	2.02 (0.44)	1.87 (0.71)	−0.15 (0.34), (−0.37, 0.08)	−0.44 (−0.73, −0.14)
95th BMI percentile (%)	119.84 (21.36)	117.38 (26.42)	−2.46 (7.02), (−7.18, 2.26)	−0.35 (−0.55, −0.15)
Body fat (%)^a^	41.71 (5.94)	40.83 (6.39)	−0.88 (2.54), (−2.83, 1.07)	−0.35 (−0.66, −0.03)
Systolic BP (mm Hg)^a^	121.00 (14.47)	122.11 (12.86)	1.11 (11.23), (−7.52, 9.75)	0.10 (−0.44, 0.63)
Diastolic BP (mm Hg)^a^	77.94 (11.07)	78.39 (11.95)	0.44 (11.30), (−8.24, 9.13)	0.04 (−0.60, 0.68)
Health‐related behaviors				
Healthy eating index	53.82 (13.76)	50.68 (14.03)	−3.15 (17.14), (−14.66, 8.37)	−0.18 (−0.92, 0.55)
Minutes in MVPA/day	3.35 (3.17)	2.58 (2.23)	−0.77 (3.29), (−2.98, 1.43)	−0.24 (−0.94, 0.47)
Minutes sedentary/day	696.11 (87.68)	740.44 (132.21)	44.34 (140.00), (−49.72, 138.39)	0.32 (−0.42, 1.06)
Hours asleep/day	7.99 (1.50)	7.20 (1.62)	−0.79 (2.49), (−2.46, 0.88)	−0.32 (−1.27, 0.63)
Psychological factors				
Quality of life	63.14 (8.80)	67.89 (5.42)	4.74 (6.67), (0.26, 9.23)	0.71 (0.13, 1.29)
Psychological flexibility	88.18 (16.00)	75.27 (19.58)	−12.91 (15.08), (−23.04, −2.78)	−0.86 (−1.47, −0.24)
Depression	22.82 (11.29)	14.27 (8.08)	−8.55 (9.89), (−15.19, −1.90)	−0.86 (−1.56, −0.17)
Perceived stress^b^	24.20 (4.32)	24.30 (4.06)	0.10 (6.24), (−4.37, 4.57)	0.02 (−0.91, 0.94)

^a^ Based on *n* = 9 with complete data due to COVID‐19.

^b^ Based on *n* = 10 with complete data.

### 3.4 Exploratory outcomes

Reductions were observed in the percentage of the 95th BMI percentile (mean: −2.46 ± 7.02, 95% CI [−7.18, 2.26], *d* = −0.35 [−0.55, −0.15]) and percent body fat (mean: −0.88 ± 2.54, 95% CI [−2.83, 1.07], *d* = −0.35 [−0.66, −0.03]). Blood pressure remained similar from baseline to postintervention.

Small reductions in minutes engaging in MVPA (mean: −0.77 ± 3.29, 95% CI [−2.98, 1.43], *d* = −0.24 [−0.94, 0.47]) and hours spent sleeping (mean: −0.79 ± 2.49, 95% CI [−2.46, 0.88], *d* = −0.32 [−1.27, 0.63]) were observed. Small increases in inactive minutes (mean: 44.34 ± 140, 95% CI [−49.72, 138.39], *d* = 0.32 [−0.42, 1.06]) were noted while HEI remained similar at postintervention relative to baseline.

Adolescents reported medium increases in perceived QOL (mean: 4.74 ± 6.67, 95% CI [0.26, 9.23], *d* = 0.71 [0.13, 1.29]). Large increases in psychological flexibility, an ABT process measure (mean: −12.91 ± 15.08, 95% CI [−23.04, −2.78], *d* = −0.86 [1.56, −0.17]), and decreases in depression (mean: −8.55 ± 9.89, 95% CI [−15.19, −1.90], *d* = −0.86 [−1.56, −0.17]) were reported. No changes were observed in reported levels of perceived stress from baseline to postintervention.

## 4 DISCUSSION

Utilizing an adolescent‐engaged approach for an ABT healthy lifestyle intervention for diverse adolescent cisgender girls with OW/OB,^42^ participants achieved metrics demonstrating acceptability related to recruitment and retention, and clinically significant decreases in BMI Z‐score were observed. Improvements in obesity‐related markers included reduced percentage of the 95th BMI percentile and percent body fat. The data also demonstrate medium to large improvements for psychological factors including perceived QOL, psychological flexibility, and depression.

Given attrition rates in youth obesity interventions range between 20% and 40%,^27^ this feasibility study demonstrated excellent retention. High levels of adherence were also observed, with over 90% of completers attending all 15 sessions and the postintervention assessment. These data are consistent with a pilot family‐based ACT obesity intervention for adolescents (*n* = 7 parent‐adolescent dyads) by Tronieri et al.,^20^ which indicated high acceptability and had 14.3 percent attrition.^20^ However, the intervention conducted by Tronieri et al.^20^ differed from our work in that it included parents and adolescents aged 12–17 with obesity only, enrolled both males and females, and did not include formative work (e.g., focus groups) or an adolescent‐engaged approach in curriculum development.

A clinically significant change in the secondary outcome of weight status using a benchmark of BMI Z‐score ≥−0.15 was hypothesized, as this decrease has been shown to lead to improvements in ≥1 CVD risk factor.^29^ The observed decrease of −0.15 in BMI Z‐score notes the occurrence of this clinically significant change, in parallel to a decrease of −2.46 in percentage of the 95th percentile and −0.88 in percent body fat. This is important as data have demonstrated nontrivial errors in BMI or BMI Z‐score in adolescents with obesity, particularly those with severe obesity.^43^ However, only three completers reduced their BMI Z‐score by more than −0.15, suggesting that this may not result in clinically significant changes in BMI Z‐score for all adolescent cisgender girls, which would have implications for considering different treatment approaches. Despite this, we did see a clinically significant decrease in BMI Z‐score ranges, which changed from 1.33 to 2.57 at baseline down to 0.23–2.59 at posttreatment. It is possible that individuals with BMI Z‐scores in the highest levels (e.g., BMI Z‐score ≥2.5) would need access to additional treatment modalities (e.g., antiobesity pharmacotherapy or bariatric surgery), along with the ABT intervention, to see clinically meaningful change. Further research is needed to examine overall efficacy, but also to identify predictors of being a responder versus non‐responder to an ABT intervention. However, observed parallel improvements in BMI Z‐score and reduced percentage of the 95th BMI percentile in the total sample of completers, along with more robust measures of change in body composition, provide preliminary evidence that an ABT healthy lifestyle intervention has the potential to improve obesity‐related health markers.

Though exploratory in nature, several psychological improvements were observed. Specifically, large improvements in psychological flexibility and experiential avoidance were presented, indicating improved aspects of self‐regulation. This aligns with previous ABT work conducted in adults that has shown increases in psychological flexibility related to eating and physical activity.^10^, ^11^, ^46^ Large improvements were also noted for adolescent depression, and medium increases were observed in perceived QOL. This suggests that ABT was beneficial not only for obesity‐related factors, but also improved aspects of mental health and QOL. This is consistent with previous ACT studies in adolescents, which have demonstrated improvements in a variety of mental health aspects, including depression and stress.^19^

Obesity‐related measures and psychological improvements observed following this 6‐month ABT healthy lifestyle intervention may be a result of the significant focus placed on self‐regulation and mental health. Adolescents with obesity are at increased risk for development of lower body image, symptoms of depression, and disordered eating behaviors when compared to those of healthy weight, further highlighting mental health as an essential component of this intervention.^45^ Adhering to dietary intake and physical activity prescribed for weight loss is exceptionally difficult for adolescents due to the interaction of pervasive cues, both physiologic (e.g., metabolic drive to maintain adiposity stores) and environmental (e.g., omnipresence of highly palatable, energy dense food).^23^ Resisting impulses or drives to consume excess calories and expend less energy requires self‐regulation skills that are actively being developed during adolescence.^13^, ^24^ While youth with obesity may demonstrate less self‐regulation than normal weight youth,^46^, ^47^ adolescence demonstrates a flexible period that opens opportunities for learning and adaptation.^13^ The limited success of adolescent weight loss interventions to date may result from limited or underdeveloped self‐regulation skills.^4^ Indeed, limited self‐regulatory skills are predictive of weight gain.^46^ Compared with those of high self‐regulation, youth with low self‐regulation have demonstrated the highest BMI Z‐scores and the most rapid gains in BMI Z‐score when assessed over a 9‐year period.^46^ Given that biological and environmental cues cannot be fully prevented or managed, and that challenging internal experiences often arise during behavioral interventions (e.g., perceived loss of pleasure, behavioral fatigue, etc.), self‐regulation skills learned through an ABT intervention may be especially critical for adolescents.

The exploratory analysis of health‐related behaviors herein indicated no evidence that the ABT intervention produced improvements in dietary patterns as measured by HEI, MVPA, sedentary time, or sleep. In fact, modest changes opposite of the expected direction were observed for some variables. Given that clinically significant changes in obesity‐related risk factors were observed, the most parsimonious explanation may be that measurement error occurred when assessing health‐related behaviors, or that an uncaptured factor reduced the validity of these measures, as it is unlikely that objective changes in obesity‐related variables would be observed without parallel changes in energy expenditure or dietary factors. Of note, HEI change was the only dietary measure, given the non‐trivial errors associated with self‐report of energy intake.^48^ It is possible, however, that energy intake was decreased despite no significant change in HEI, which measures overall diet quality. It is also possible that participants at baseline were eager to “impress” or “please” the research team, inflating baseline self‐report data. There is also potential that the ABT intervention did not have the intended effect on health‐related behaviors, and further research in a fully powered sample is needed. It is important to note that for a subset of the participants, the 2020 COVID‐19 pandemic factored into implementation and four participants were required to transition from in‐person to remote intervention delivery for Sessions 9–15. Research conducted among participants involved in health‐related research during the 2020 COVID‐19 “stay‐at‐home‐orders,” coinciding with this study timeline, demonstrated significant experiences of adverse mental health and ability to adhere to behavioral recommendations of an intervention.^22^ These findings would suggest a potential underestimation of the effect size of ABT on obesity‐related measures, health‐related behaviors, and psychological outcomes. With consideration of demonstrated attrition issues in previous studies, as well as the intensive time commitment demanded of behavioral interventions, remote delivery of behavioral obesity research warrants further study.

Conducting an ABT healthy lifestyle intervention for adolescent cisgender girls with OW/OB from diverse backgrounds (both racial/ethnic and socioeconomic diversity) is feasible. Given the high acceptability of this intervention, and the promising preliminary outcomes, a fully powered, randomized controlled trial (RCT) is warranted to assess the efficacy of an ABT healthy lifestyle intervention compared to a standard care of SBT intervention in adolescents. An efficacy trial would allow for assessment of the extent to which ABT can improve weight status, cardiometabolic risk factors, and psychosocial functioning among adolescent cisgender girls with OW/OB. It would also allow for identification of the moderating effects of demographic (race/ethnicity, age, socioeconomic status) and psychological factors via statistical interactions in a scientifically rigorous manner. It is important to acknowledge limitations and consider that no control group was included, thus changes in outcomes could be due to time or social desirability bias, that the results are only generalizable to cisgender adolescent girls, and that the sample size was not sufficient to assess differences by race/ethnicity, age, or socioeconomic status. Furthermore, regression to the mean may account for changes in scores at post‐intervention, providing further support to test these hypotheses in a fully powered RCT. The instructor also had a doctoral degree in nutrition sciences and was a registered dietitian. Further research should explore the feasibility and efficacy of an interventionist with less specialized training (e.g., community health workers), which would allow for greater implementation and scalability.

Preliminary data suggest an ABT healthy lifestyle intervention among adolescent cisgender girls from diverse racial/ethnic backgrounds is an acceptable intervention. Preliminary data suggest ABT can result in clinically significant improvements in obesity‐related measures, and notable improvements in psychological factors, but further research is needed with a fully powered RCT. These data, combined with the pilot work of Tronieri et al.,^20^ suggests that an acceptance‐based approach for behavioral weight loss is acceptable for adolescents with OW/OB.

## CONFLICT OF INTERESTS

Michelle I. Cardel has conducted paid consulting for WW and unpaid consulting for NovoNordisk. The other authors declare that there are no conflict of interests.

## AUTHOR CONTRIBUTIONS

Michelle I. Cardel, David M. Janicke, and Meghan L. Butryn conceived study. Michelle I. Cardel, Alexandra M. Lee, Faith Newsome, and Darci R. Miller carried out study. Xiaofei Chi and Matthew J. Gurka analyzed data. Angelina Bernier andLindsay Thompson were consulted on study design and data interpretation. All authors were involved in writing the manuscript and had final approval of the submitted and published versions.
